# Conversion events in gene clusters

**DOI:** 10.1186/1471-2148-11-226

**Published:** 2011-07-28

**Authors:** Giltae Song, Chih-Hao Hsu, Cathy Riemer, Yu Zhang, Hie Lim Kim, Federico Hoffmann, Louxin Zhang, Ross C Hardison, Eric D Green, Webb Miller

**Affiliations:** 1 Center for Comparative Genomics and Bioinformatics, Pennsylvania State University, University Park, PA 16802 USA; 2 Computational Biology Branch, National Center for Biotechnology Information, National Library of Medicine, National Institutes of Health (NIH), Bethesda, MD, 20892 USA; 3 Department of Biochemistry and Molecular Biology, Mississippi State University, Mississippi State, MS 39760, USA; 4 Department of Mathematics, National University of Singapore, 117543, Singapore; 5 NIH Intramural Sequencing Center (NISC) and Genome Technology Branch, National Human Genome Research Institute, National Institutes of Health, 50 South Drive, Bethesda, MD 20892 USA

## Abstract

**Background:**

Gene clusters containing multiple similar genomic regions in close proximity are of great interest for biomedical studies because of their associations with inherited diseases. However, such regions are difficult to analyze due to their structural complexity and their complicated evolutionary histories, reflecting a variety of large-scale mutational events. In particular, conversion events can mislead inferences about the relationships among these regions, as traced by traditional methods such as construction of phylogenetic trees or multi-species alignments.

**Results:**

To correct the distorted information generated by such methods, we have developed an automated pipeline called CHAP (Cluster History Analysis Package) for detecting conversion events. We used this pipeline to analyze the conversion events that affected two well-studied gene clusters (α-globin and β-globin) and three gene clusters for which comparative sequence data were generated from seven primate species: CCL (chemokine ligand), IFN (interferon), and CYP2abf (part of cytochrome P450 family 2). CHAP is freely available at http://www.bx.psu.edu/miller_lab.

**Conclusions:**

These studies reveal the value of characterizing conversion events in the context of studying gene clusters in complex genomes.

## Background

Recent comparative genomics studies have revealed how the human genome has been shaped by various evolutionary forces. Some regions in the human genome are strongly conserved among many mammalian species [1,2], while others have seen accelerated change in the human lineage compared to other species [3,4]. Some regions, the so-called "gene clusters", are composed of multiple similar copies of gene-containing segments in close proximity. Because such structurally complex regions have been implicated in human genetic diseases, their study has become of great interest. For example, the number of gene copies in the CCL cluster influences susceptibility to HIV [5], the IFN cluster is associated with sarcoidosis [6], and the CYP2abf cluster is implicated in lung cancer [7].

**Figure 1:**
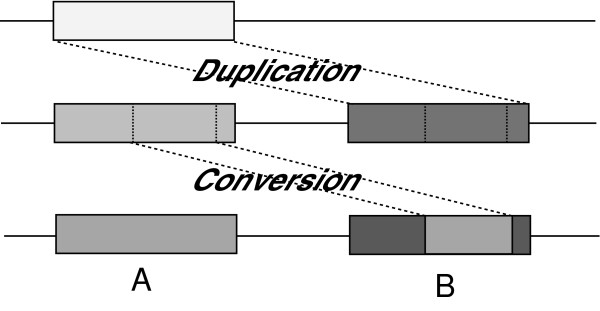
**Depiction of a conversion event**. First, a genomic region A is copied into a new position by a duplication event, forming an additional paralogous copy B. Over time, the two paralogs (A and B) diverge due to small mutations. Then, a conversion event overwrites a portion of one paralog (e.g. B) with the corresponding portion from the other paralog, so that A and B become more similar in that sub-region than elsewhere.

Analyzing the evolution of biomedically relevant gene clusters can inform studies aiming to discover the molecular mechanisms underlying their genetic disease association (e.g. [8]). However, gene clusters are difficult to analyze because they contain multiple similar genomic regions, and tend to have complex evolutionary histories involving a variety of large-scale mutation events (such as duplications, deletions, inversions, and conversions). One of the key problems in analyzing gene clusters lies in distinguishing between orthologs, defined as genes that derive from speciation events, from paralogs, defined as genes that derive from duplication events. Traditional efforts to trace the relationships among these regions have focused on constructing a phylogenetic tree or a multiple alignment of homologous sequences, but these approaches suffer from uncertainty problems whereby different methods can produce substantially different results [9]. Even when using a single method, the tree topology of a sequence dataset can change depending on which region is selected [10]. One of the main factors confounding such evolutionary analyses is the phenomenon of conversion between paralogs (a.k.a. non-allelic or ectopic conversion), where sequence from one region overwrites part of a similar paralogous region via the recombinational machinery (Figure 1). This is sometimes called "gene conversion", though it does not necessarily involve any genes. It is typically caused by DNA double-strand breaks or by a double Holliday junction dissolution mechanism [11]. Previous genome-wide studies of conversion revealed that such events have occurred quite often (e.g. 7.5% of all paralogous pairs in the mouse genome [12] and 13.5% in the human genome [13]). Moreover, some of these paralogs have undergone recurring conversion events, complicating matters even further.

Many computational methods have been devised for detecting gene conversion (e.g. [12,14-16]). According to our evaluation study [17], the method we developed [12] outperformed the others tested when applied to gene cluster data and both sensitivity and false discovery rate were taken into account. In addition, the other methods for detecting converted regions pose the inconvenience of requiring a multiple sequence alignment for each set of homologous sequences. Here, we report the development of an easy-to-use software package called CHAP (Cluster History Analysis Package) for detecting conversion events using gene cluster sequences from multiple species. CHAP uses the conversion-detection method from [12], which is based on a statistical test proposed by [18]. The CHAP package includes procedures for identifying all pairwise orthologs required by the conversion-detection method, utilizing an extension of our CAGE program [19]. Thus, users only need to prepare sequence datasets for their gene clusters of interest, and provide an overall phylogenetic tree for the species involved. The package also includes an extended version of our Gmaj viewer [20], which provides a convenient way to examine the detected conversion events. The entire CHAP package is available for free download from our website at http://www.bx.psu.edu/miller_lab. Users will also need the well-known RepeatMasker program [21], which is available from http://www.repeatmasker.org.

CHAP includes the following differences from our previous program [12]. (1) CHAP focuses on analyzing gene clusters rather than entire genomes. In particular, it incorporates an ortholog identifier specifically designed for dealing with the clusters' complex evolutionary histories [19], whereas the program from [12] relies on external, generic ortholog assignments. (2) It detects conversion events in multiple related species at once, not just a single primary species as in [12]. (3) It leverages the multi-species conversion data to estimate the time of the events in the species tree. (4) It includes an additional, alternative test criterion that enables it to detect conversions covering entire paralogs, which were missed by [12]. (5) It provides a visualization tool to help users easily investigate the conversion results.

We used CHAP to analyze conversion events in five primate gene clusters. This involved using a combination of sequences available from public sources and newly generated ones (see Additional file 1, Table S1 in the supplement for the GenBank accession numbers of the new sequences). The clusters were chosen based on their relevance to human health, recent duplications in the human lineage, and small size: β-globin (hg19.chr11:5,224,419-5,314,419), α-globin (hg19.chr16:190,000-235,000), CCL (chemokine ligand; hg19.chr17:34,310,693-34,812,885), IFN (interferon; hg19.chr9:21,058,760-21,481,698), and CYP2abf (part of cytochrome P450 family 2; hg19.chr19:41,324,635-41,712,359).

## Results and discussion

### Overview of detecting conversion events affecting gene clusters

A conversion event produces a within-species alignment where one part is better conserved than other parts. To distinguish this from variation caused by natural selection, our method compares the alignment of paralogs with that of an ortholog in another species to factor out the effects of purifying selection. The use of sequence from an outgroup species that split from the species of interest after the duplication that gave rise to the paralogs, but before the conversion event, can help to illuminate the situation.

**Figure 2:**
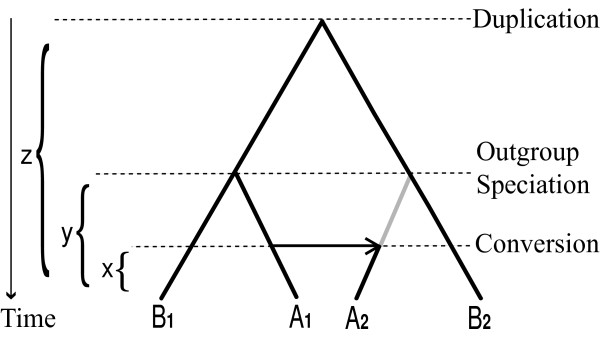
**Condition for detecting a conversion event**. Suppose that conversion, outgroup speciation, and duplication events occurred *x, y*, and *z *years ago, respectively (*x *<*y *<*z*), and the events formed regions A_1 _and A_2 _in primary species A and regions B_1 _and B_2 _in the outgroup species B (note that in this type of tree, the nodes represent homologous sequence segments). We observe that the paralogous sequences A_1 _and A_2 _are more similar than would be expected without a conversion, i.e. the evolutionary distance between them is less than that between orthologs A_1 _and B_1_, though the distance between paralogs B_1 _and B_2 _is greater.

To illustrate the general idea, suppose these conversion, outgroup speciation, and duplication events occurred *x, y*, and *z *years ago, respectively (*x < y < z*), forming regions A_1 _and A_2 _in primary species A and regions B_1 _and B_2 _in outgroup species B (Figure 2). Thus before the conversion, A_1 _is orthologous to B_1 _and A_2 _to B_2_, while A_1 _is paralogous to A_2 _and B_1 _to B_2 _by the definitions of orthology and paralogy [22,23]. Let dist(X, Y) denote the evolutionary distance between two regions X and Y. The distance between species A and B is 2*y *because A and B split *y *years ago, so in the absence of conversion, we expect dist(A_1_, B_1_) = 2*y *and dist(A_2_, B_2_) = 2*y*. Similarly, for the paralogs we expect dist(B_1_, B_2_) = 2*z *and dist(A_1_, A_2_) = 2*z*. However, the conversion event causes dist(A_1_, A_2_) = 2*x *and dist(A_2_, B_2_) = 2*z *instead. Whenever we observe two paralogous regions A_1 _and A_2 _and an outgroup species B such that dist(A_1_, B_1_) < = dist(B_1_, B_2_) and dist(A_2_, B_2_) < = dist(B_1_, B_2_), but dist(A_1_, B_1_) > dist(A_1_, A_2_) and dist(A_2_, B_2_) > dist(A_1_, A_2_) when A_1 _is orthologous to B_1 _and A_2 _to B_2_, then we infer a conversion event between A_1 _and A_2_. Note that our statistical method was designed for unequal rates of evolution as well as a molecular clock, although we illustrate only the latter case here for simplicity.

**Figure 3:**
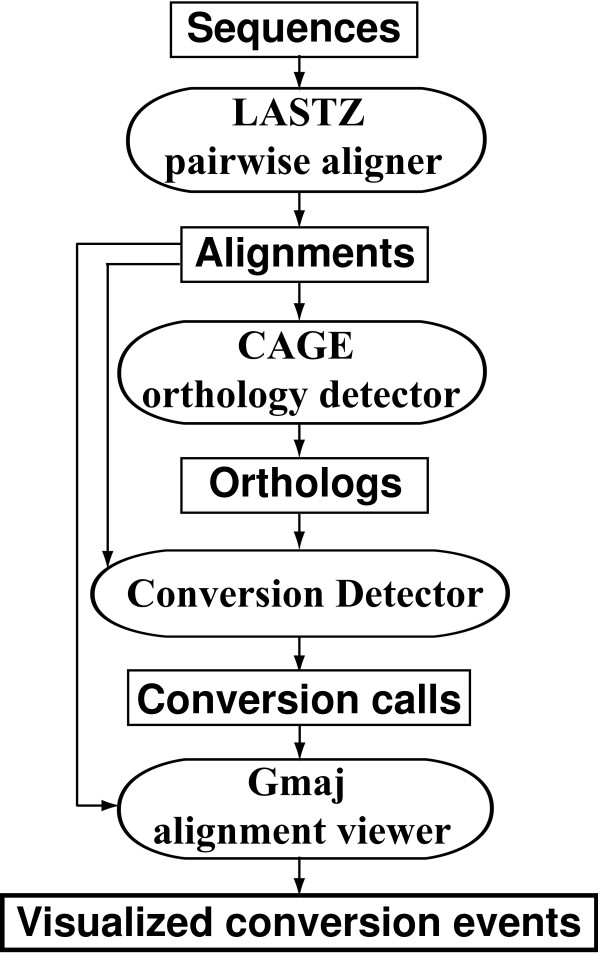
**Overview of the CHAP pipeline**. Rectangular boxes represent data files or graphical display of results, while ovals denote software programs.

**Figure 4:**
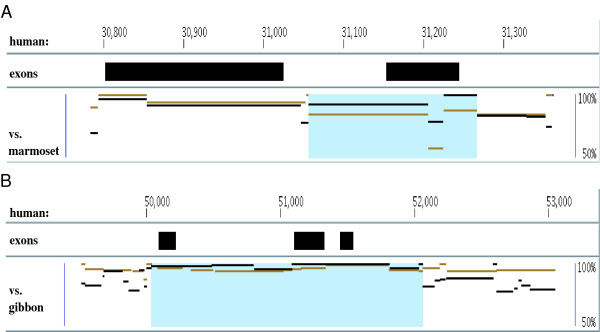
**Examples of conversion events in the human β-globin cluster**. Blue underlays in the plots represent conversion regions. Black lines show sequence similarities between a human region and its human paralog, and brown lines between the human region and its ortholog in the outgroup species. In each plot, the y-axis corresponds to the percent identity of the two sequences (50%-100%). Note how the black paralogs lie above the brown orthologs in the converted region, but fall below them in the flanking regions. (A) depicts a conversion event in the first exon and intron of the human δ gene, copied from the human β gene (standard coordinates for this human chromosome have the genes of the β-globin cluster transcribed from right to left). This event was detected using the marmoset ortholog of human δ. (B) shows a converted region covering the human γ2 gene. Its paralog is γ1 in human, and the gibbon ortholog of human γ2 was used for this conversion observation.

**Figure 5:**
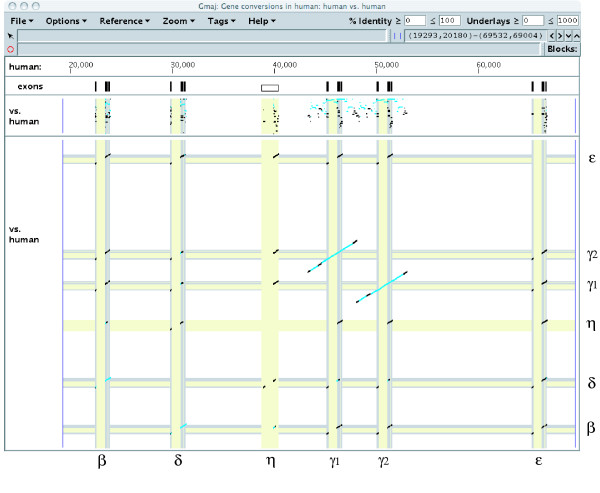
**Detected conversion events in the human β-globin cluster**. The conversion events detected by CHAP in the human β-globin cluster are depicted, as displayed using Gmaj. The upper plot panel displays a pip (percent identity plot), similar to those in Figure 4. The lower one shows the dot-plot for a self-alignment of the human cluster. Each conversion event is marked in blue on a local alignment of two paralogous regions. Our results show conversion events in all of the genes, and also the pseudogene η (some are very small and difficult to see at this scale, but are evident when zooming in with Gmaj). The conversion between the γ1 and γ2 genes covers their non-genic flanking regions as well. Note that repeated conversion events can occur in the same paralogous pair with different boundaries. Here, the δ-β conversion appears to cover both the first and second exons (which is more than in Figure 4A) because of an older, wider conversion between these paralogs that was detected when galago and dog were used as outgroups.

**Figure 6:**
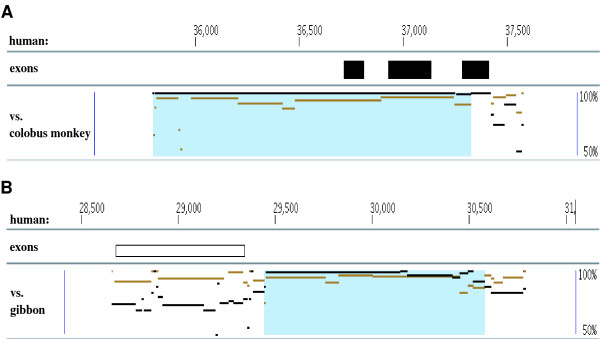
**Examples of conversion events in the human α-globin cluster**. As in Figure 4, the blue underlays represent regions involved in conversions (either as donor or recipient), while the black and brown lines plot the percent identity between the human paralogs and between one paralog and its ortholog in the indicated outgroup species, respectively. (A) depicts a region surrounding the α1 gene whose paralog contains α2. Here, the displayed region was the donor, and the conversion covers most of the paralog. The lack of unconverted flanking region on the left prevents CHAP's original detection criterion from working in this case, but the event is detected using a new alternative criterion. (B) shows a conversion observation between paralogs containing the α3 pseudogene and the α2 gene. In this case, the paralog containing the pseudogene (shown as an empty box in the exons panel here) was the recipient, but the converted region did not actually include any parts of the α2 gene.

**Figure 7:**
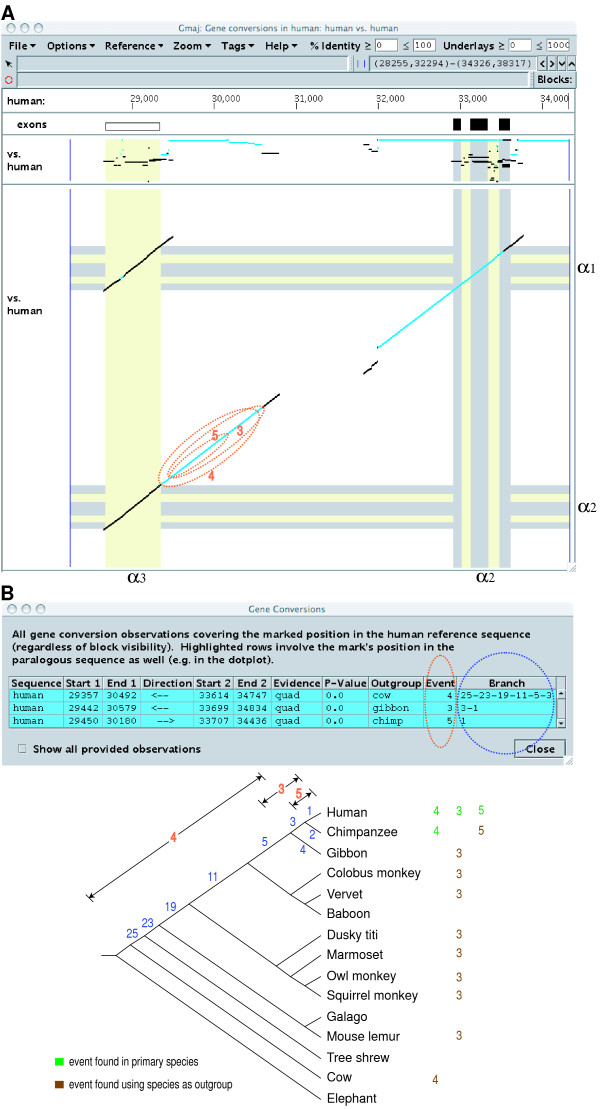
**Timing of repeated conversion events in the human α-globin cluster**. Three sequential events occurred between paralogs containing the α3 pseudogene and the α2 gene. (A) The regions involved in each conversion are marked here with orange ovals on the self-alignment dot-plot for the human cluster, and labeled with their corresponding event id numbers. (B) The table displayed by Gmaj shows a summary of these events. The Event column (highlighted here with an orange oval) shows the assigned event id, and the Branch column (blue oval) indicates the estimated conversion time as a sub-lineage of numbered tree edges. The tree and colored numbers added here illustrate how to interpret the table and what information was used to infer the conversion time. Numbers in green are the ids of events found in the particular species (i.e. as primary), while those in brown indicate events found using that species as outgroup. Primary species are used to estimate the lower bound for the conversion time; e.g. we inferred that event 4 occurred before the split of human and chimpanzee because we observed this event in both species (in paralogous pairs that are orthologous to each other). In contrast, an outgroup species used for detecting an event provides evidence for the upper bound of its conversion time, i.e. the event occurred after the split of the primary and outgroup species. For example, event 5 must have happened after the separation of human and chimpanzee because it was detected when using chimpanzee as the outgroup and human as the primary species. CHAP does not draw any inference from negative results (lack of detection), since that may be due to other factors such as missing sequence.

**Figure 8:**
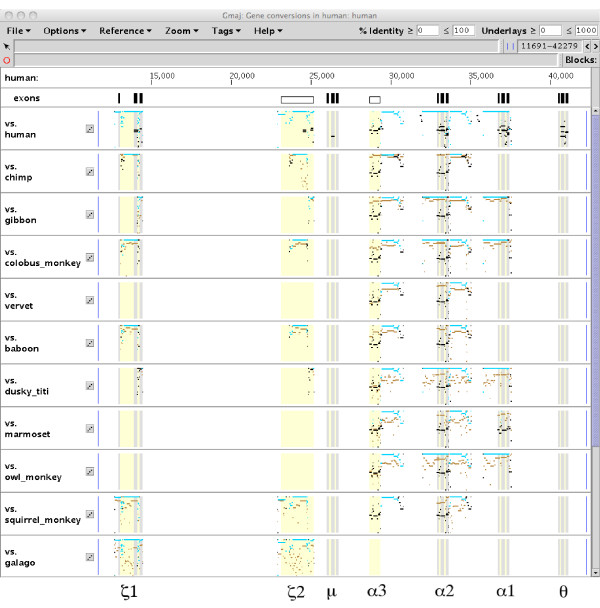
**Detected conversion events in the human α-globin cluster**. The human α-globin cluster contains five genes (ζ1, μ, α2, α1, and θ) and two pseudogenes (ζ2 and α3) (yellow). Each gene comprises three exons (gray) and two introns (yellow). The first panel plots the percent identities of the aligned human paralogs. Blue segments indicate conversion events in the human lineage. The other panels are "evidence plots" similar to Figures 4 and 6, each focusing on conversion observations detected using a particular outgroup. They recapitulate the relevant subset of paralog alignments from the first panel (black/blue), superimposed on the alignments to the orthologs in the outgroup species (brown) for comparison.

The CHAP pipeline takes as input a set of FASTA sequences from a number of species for a particular gene cluster, together with a phylogenetic tree describing the overall relationships among the species. With a single command, it detects conversion events in all of the species using the others as outgroups. Figure 3 illustrates the steps. First, all self alignments (aligning each sequence to itself) and pairwise inter-species alignments are generated (in MAF format) using the LASTZ aligner [24]. Next, the CAGE software [19] uses a combinatorial approach to identify all pairwise orthologs in the alignment data, according to the definition of orthology in [22,23]; its output is a subset of the inter-species alignments, also in MAF format. Then, our conversion detector [12] examines each pair of paralogous regions, together with their orthologs in each outgroup species, and performs several statistical tests to infer conversion events (see Methods). The primary output from this step is a tab-separated text file listing the conversion observations for each combination of reference and outgroup species, which are grouped into unique events (since the same event may be observed in multiple species and/or by using multiple outgroups) and localized to a particular sub-lineage in the phylogenetic tree. Finally, a second command invokes an extended version of our Gmaj viewer [20] to visualize the detected conversion observations in a chosen species using an interactive, graphical display. Figures 4, 5, 6, 7, 8 and 12 show examples of conversion events detected and visualized by CHAP.

### Conversion events affecting the primate β-globin cluster

The human β-globin cluster is composed of five genes (listed 5'→3'): HBE (ε), HBG2 (γ2), HBG1 (γ1), HBD (δ), and HBB (β). Each gene contains three exons separated by two introns. The human cluster also includes a well-known pseudogene HBH (η) located between the γ1 and δ genes. The human sequence and annotation data for this cluster were obtained from the UCSC Genome Browser (genome.ucsc.edu). We downloaded the sequences of 11 other primate species from the GenBank and Ensembl databases, and obtained gene annotation information for the non-human primate species using the GeneWise program [25]. The non-human primate species were used by the CHAP pipeline as outgroups to detect conversion events in the human lineage. We also used dog and elephant sequences from the UCSC Genome Browser as additional outgroup species.

Evidence suggests that the common ancestor of the primates had a structure of 5'-ε-γ-η-δ-β-3' in this cluster [26-28]. Except for γ1/γ2, duplication events among these genes predate the split of primate species [29], i.e. the sequence similarities of two paralogs among those genes are usually lower than the similarities to their orthologs in other primate species [8]. However, Figure 4A shows a case where a region containing the first exon and part of the first intron of the human δ gene has a higher sequence similarity to its paralog β than to its marmoset ortholog, while the ortholog pair shows the expected higher similarity levels than the paralogs in the flanking regions and other parts of human δ. CHAP detected that the blue-shaded region of human δ was copied from human β via a conversion event. This conversion inference is strongly supported by a *P*-value of 2.31 * 10^-5^; it also agrees with other studies of evolutionary relationships in the β-globin cluster (e.g. [28,30]).

Previous studies on the duplication of the γ1 and γ2 genes have traced that tandem duplication to a time before the split of the catarrhine primates (humans, apes, and Old World monkeys) although sequence comparison of γ1 and γ2 shows a high similarity level at about 98% identity (e.g. [31]). This has been reported as an example of gene conversion [11,32]. CHAP detected a conversion event in the human γ2 gene copied from γ1, e.g. when using gibbon as the outgroup (Figure 4B). Our result indicates that this conversion includes the flanking regions of the two γ genes as well as their exons and introns, in total involving approximately 2000 bp. In addition to these two main conversion events in the human β-globin cluster, there are several minor ones as well (Figure 5).

### Conversion in the α-globin cluster and events covering entire paralogs

The human α-globin gene has the same origin as the β-globin gene, i.e., both were generated via duplication about 450 million years ago from a common ancestral globin gene [33]. Since then, they have formed their current clusters independently [28]. Similar to the β-globin cluster, the human α-globin cluster comprises five genes (listed 5'→3'): HBZ-T1 (ζ1), HBK (μ), HBA-T2 (α2), HBA-T3 (α1), and HBQ (θ). Each gene contains three exons separated by two introns. In addition, there are two well-known pseudogenes: HBZ-T2 (ζ2) and HBA-T1 (α3). We obtained sequences and annotation information for human and 12 non-human primate species, cow, and elephant for the α-globin cluster as above for the β-globin cluster (except that pseudogene locations were inferred from the human self-alignment). We then ran the CHAP pipeline to detect conversion events in this cluster.

One of the interesting results in the human α-globin cluster is a conversion involving the α1 and α2 globin genes. The duplication giving rise to these paralogs predates the separation of the apes from New World monkeys [34]. However, the evolutionary distance between these paralogous sequences is less than that between α1 and its ortholog in colobus monkey (Figure 6A). The ortholog situation is straightforward, since the colobus orthologs that the CAGE step identified for the human α1 and α2 genes are one-to-one; i.e. CAGE does not find any post-speciation duplications involving α1 or α2 in either the human or colobus lineages, so the human paralogs are mapped to single, distinct regions in colobus. Nevertheless, the original criterion used in [12] cannot detect the conversion event shown in Figure 6A because that criterion requires switch points of percentage identity differences between paralogs and orthologs, as shown in Figure 4 and Figure 6B. The reason that such pattern switches do not appear in Figure 6A is that nearly the entire sequence is involved in the conversion, so there are insufficient flanking regions for performing the test. To detect such events, we added a new criterion to the conversion detector in our CHAP pipeline that does not require flanking regions (see Methods). As a result, CHAP was able to detect this event, where according to the colobus outgroup, 1526 bp of the human paralog containing α1 overwrote the corresponding part of the α2 paralog. This observation is supported by a *P*-value of 5.29 * 10^-20^. The same event is also observed using other outgroups, though some of them show the opposite direction of conversion and different endpoints for the converted region.

Another interesting conversion occurred between paralogs containing the human α2 gene and α3 pseudogene (Figure 6B). The duplication forming α2 and α3 predates the separation of the simian primates from prosimians [34]. Since the conversion can be observed using gibbon as the outgroup sequence, we conclude that it occurred after the separation of hominids from other apes. Although the human α3 region is a pseudogene, its 3'-flanking region still has a sequence similar to that of the protein-coding gene α2 due to the recent conversion event. We speculate that this conversion may play a role in conserving some function associated with the 3'-flanking regions of α2 and α3. The α3 pseudogene is present as a pseudogene in several species, which suggests that it is somewhat important, given that it lost function several million years ago yet is still around in some of the species examined. Further, its ortholog in prosimians is an active gene.

The conversion events detected here include those originally described for the duplicated α-globin genes in humans [35]. Also, the "gradient of gene conversion" [36] is apparent in the declining percent identity of the converted DNA segments in the 3' flanking regions of the α3 and α2 genes (Figure 7). This gradient was attributed to repeated gene conversions over evolutionary time. By examining the patterns of conversion detected using outgroups over increasing phylogenetic distance, CHAP inferred three different conversion events in the 3' flanking regions of the α3 and α2 genes, and estimated the time of each event as illustrated in Figure 7B. For instance, event 5 in Figure 7 is a recent conversion that happened after the split of human and chimpanzee, while event 4 occurred before their separation. Note that the conversion time for event 4 is difficult to determine precisely because we do not have sufficient information from many of the species (mainly due to missing sequences), whereas event 3 has eight outgroup results supporting the inference that it followed the separation of human and gibbon.

All of the conversion events detected by the pipeline in the human lineage of the α-globin cluster are shown in Figure 8, which displays them on a human self-alignment together with inter-species alignments between human and the outgroups used for the event detection. In total, 11 conversion events were detected, all involving paralogs containing ζ1, ζ2, α3, α2, and/or α1.

### Summary of conversion events in five human gene clusters

**Figure 9:**
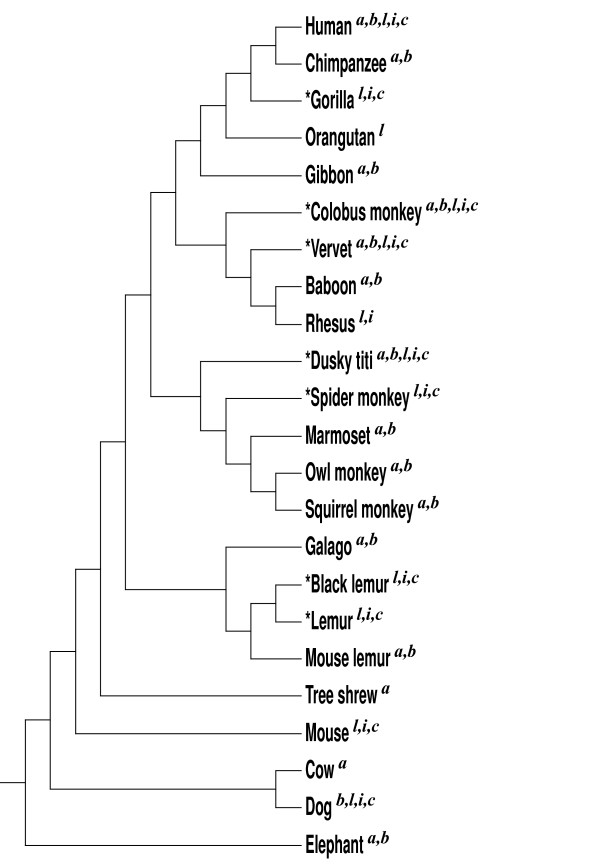
**Species used for the analysis of conversion events in the five gene clusters**. The phylogeny for these species was derived from [53,54]. Those used for the analysis in each gene cluster are marked with superscripts as follows: ^a^α-globin, ^b^β-globin, ^l^CCL, ^i^IFN, and ^c^CYP2abf. "*" indicates the seven species for which sequence data was newly generated for the CCL, IFN, and CYP2abf clusters. The human, mouse, cow, dog, and elephant sequences were downloaded from the UCSC Genome Browser, and the other primates from GenBank and Ensembl.

In addition to the β-globin and α-globin clusters, we obtained sequences for three more gene clusters: CCL, IFN, and CYP2abf. For these studies, comparative sequence data were generated from seven additional primate species in these clusters, indicated by "*" in Figure 9.

**Figure 10:**
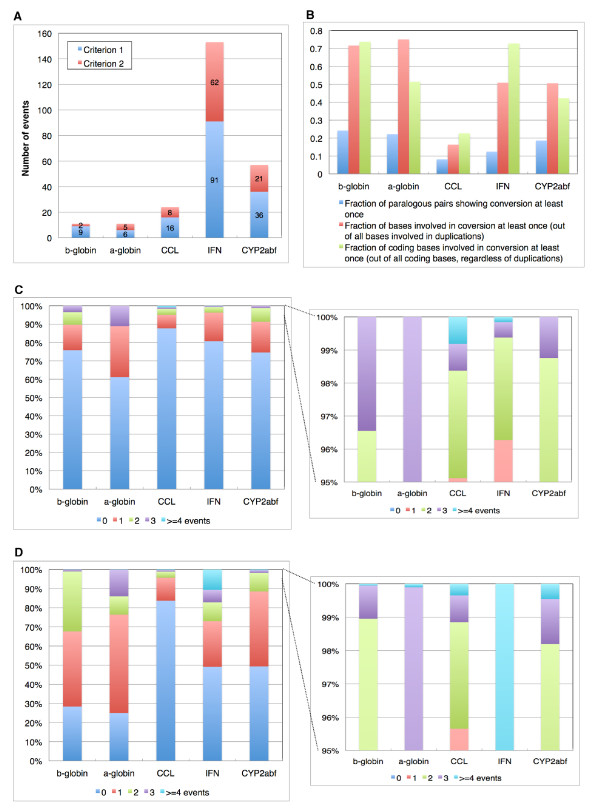
**Summary of detected conversions in the five gene clusters**. (A) Number of conversion events via criterion 1 and criterion 2. (B) Conversion frequencies in the general paralogous sequences and protein-coding exons. (C) Fraction of paralogous pairs by their number of conversion events, out of all paralogous sequence pairs. (D) Fraction of bases by their number of conversion events (involved as either source or target), out of all bases involved in duplications.

We analyzed conversion events in the human lineage for these five clusters using the CHAP package. As shown in Figure 10, conversions are quite frequent in all of these clusters. Here, we classify the events into two categories: criterion 1 or 2. Criterion 1 means that the event was detected using the original triplet or quadruplet tests from [12], while criterion 2 is the new method for detecting events covering the entire paralogous sequence (or most of it), without sufficient unconverted flanking regions needed for criterion 1. Figure 10A shows the number of events detected by each criterion in the five clusters. Of the 256 total events, 38.3% were detected by criterion 2.

To examine the conversion frequency, we first computed the fraction of paralogous sequence pairs showing conversion, displayed in the blue bars of Figure 10B. There were a total of 975 paralogous sequence pairs in the five clusters, and of these, 194 (19.9%) experienced at least one conversion event. By this measure, the α-globin cluster is the most active of the five, with 38.9% of its pairs showing evidence of conversion.

We also computed the conversion frequency using the number of bases instead of paralogous pairs, shown in the red bars of Figure 10B. A total of 725,694 bases in the five clusters lie in duplicated regions, and 37.7% of them have been involved in a conversion at least once. This fraction is almost twice the one based on the number of pairs. In this comparison based on the number of bases, the α-globin cluster again shows the highest conversion frequency, with 75.0% of its duplicated bases involved in conversions. According to both measures, the CCL cluster experienced the least conversion activity.

In addition to the conversion frequencies in the general paralogous sequences, we also analyzed the incidence of conversion in protein-coding exons (green bars in Figure 10B). On average, nearly 50% of the coding bases were involved in at least one conversion. In the β-globin and IFN clusters, this rose to more than 70%. The frequent conversion events between the coding regions serve to homogenize paralogs within species, which could contribute to the establishment of species-specific characteristics. Furthermore, conversions that copy non-coding sequences into coding regions (e.g. as in the human CYP2A13 gene, discussed below) can introduce new functionality more quickly than point mutations, since many nucleotides are changed by a single event. IFN and CCL are involved in immune response to infection by pathogens such as viruses, bacteria, or tumor cells, and the CYP450 genes play crucial roles in the metabolism of exogenous substances such as drugs or carcinogens. Thus gene conversion may be an effective evolutionary mechanism facilitating adaptation to environmental changes.

Some paralogous pairs are involved in conversions more than once. Figure 10C shows how many pairs in each cluster experienced various numbers of conversion events. About 10% of the paralogous pairs in the β-globin and α-globin clusters had at least two sequential events, and this figure was at least 4% for the other three clusters.

We also computed the proportion of bases involved in multiple conversions, either as source or target, out of all bases in duplicated regions (Figure 10D). More than 20% of the duplicated bases in the β-globin, α-globin, and IFN clusters were involved in conversion events at least twice. In the IFN cluster, about 10% were involved in four or more events.

In addition, we identified the "hot-spot" protein-coding gene in each cluster according to the number of conversion events involving coding exons, either as donor or recipient (Table 1).

**Table 1:** Hot-spot genes for conversion events

Cluster name	Gene name	Chromosome	Coding start	Coding end	Number of events
β-globin	HBB	chr11	5,246,828	5,248,251	6

α-globin	HBZ	chr16	202,909	204,399	5

CCL	CCL15	chr17	34,324,803	34,328,531	5

IFN	IFNA14	chr9	21,239,365	21,239,934	13

CYP2abf	CYP2A13	chr19	41,594,377	41,601,846	7

The protein-coding gene in each cluster showing the most conversion events involving exons. Positions are given with respect to the UCSC hg19 assembly.

When conversions occur in protein-coding genes, the functions of the genes may be affected. For example, we detected recurrent conversions between the human β- and δ-globin genes. Evidence for a similar but presumably much more recent event has been reported in a patient exhibiting mild microcytosis, whose δ-globin gene contained part of the amino acid sequence of the β-globin gene [37]. The authors of that report hypothesized that this could have been due to a new gene conversion.

### The human CYP2A13 gene as a hot-spot for conversions in the CYP2abf cluster

**Figure 11:**
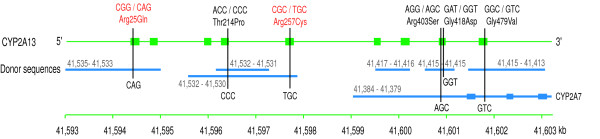
**Conversion events in the CYP2A13 gene**. The detected conversions are mapped in the human CYP2A13 gene region, hg19.chr19:41,594,377-41,601,846. Green boxes represent exons, and blue lines indicate donor sequences which correspond to the positions of converted regions in the CYP2A13 gene. The numbers on the blue lines indicate the chromosomal positions of the donors in kilobases (kb). Blue boxes represent exons in the human CYP2A7 gene, which is one of the donors. The six non-synonymous SNPs indicated by black vertical bars on the exons have mutant alleles that share the same nucleotides as the donor sequences. For example, for the Arg257Cys SNP, the mutant, minor allele is Cys (TGC) in the human population, and the donor sequence at that position is also TGC. Arg25Gln and Arg257Cys (in red) are known to be associated with metabolic activity of a tobacco-specific carcinogen.

To examine the effect of conversion events on gene function, we focused on the CYP2A13 gene from Table 1. CHAP detected seven conversion events, all overwriting parts of this gene with donor sequences from elsewhere, and together they involved all nine of CYP2A13's coding exons (Figure 11). Interestingly, except for one event the donor paralogs are entirely non-coding, and according to annotation data from NCBI, none are considered to be pseudogenes. It is somewhat surprising that the gene is still functional, in spite of being overwritten with non-coding sequences so many times.

The CYP2A13 gene encodes an enzyme which metabolizes a tobacco-specific carcinogen, NNK (4-(metholnitrosamino)-1-(3-pyridyl)-1-butanone) [38]. NNK is activated by the metabolism, leading to its carcinogenicity [39]. The gene is intensively expressed in the respiratory tract [38,40], and it has been studied as a candidate gene associated with tobacco-related cancers, such as lung cancers. In particular, the variations Arg25Gln and Arg257Cys have been shown to relate to metabolic activities involving NNK [41-43]. The minor alleles, Gln and Cys, decrease the activities of the enzyme as well as the gene expression, and could produce lesser toxicity of NNK in smokers [44].

To investigate the effects of conversions on the CYP2A13 coding sequences, we compared sites in these regions that are polymorphic in the human population against the nucleotide sequences of the donor regions. In particular, we used non-synonymous SNP sites from the NCBI dbSNP database in the converted CYP2A13 exon regions, and looked for cases where the mutant allele of the SNP matches the donor sequence of a conversion detected in this study (Figure 11). The mutant allele was determined under the assumption that the gorilla orthologous sequence is the ancestral type. Indeed, the donor sequences for the 25th and 257th amino acids of CYP2A13 do match the minor alleles, which are also the mutant alleles. We also found four other non-synonymous SNPs in this gene whose mutant alleles share the same nucleotides as the donor sequences. This frequent sharing could be explained by recurrent conversions from the donor sequences in the human population, as an alternative to point mutations alone. Thus we suggest that recent conversions, perhaps tending to recapitulate ancestral ones, may contribute to polymorphism and phenotypic variation in humans. (Note that CHAP would not detect such recent conversions here, as it was only given the reference assembly for each species.)

### Conversion in *Drosophila melanogaster *paralogs

Conversion in the *D. melanogaster *genome has been studied by many researchers (e.g. [45-47]). Approximately 7.5% of its paralogous genes have experienced conversion [46]. To explore the applicability of CHAP for studying non-mammalian sequences, we used it to analyze a gene cluster where we suspected the occurrence of conversion.

**Figure 12:**
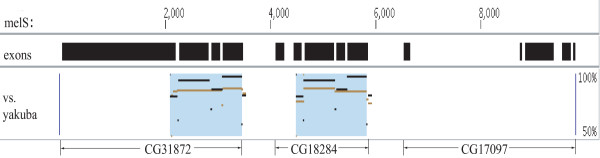
**Detected conversion event in *Drosophila melanogaster***. The conversion event between the CG31872 and CG18284 genes is visualized using Gmaj, similar to Figures 4 and 6. D. yakuba was used as the outgroup species. The blue underlay in CG31872 is the recipient, overwritten by the corresponding region in CG18284.

The genomic region dm3.chr2L:10,639,900-10,649,700 (FlyBase R5.33) [48] contains three paralogous genes (CG31872, CG18284, and CG17097), which are associated with lipase activity and lipid metabolic processes [49]. The CG18284 and CG17097 genes comprise five exons each, while CG31872 has four. We located the corresponding genomic region in D. yakuba (droYak2.chr2L:7,042,800-7,053,500) using inter-species LASTZ alignments for use as an outgroup. CHAP detected a 1354-bp conversion event between the CG31872 and CG18284 genes that involves their last four exons and three introns (Figure 12). It is interesting to note that the conversion event maintained the exon-intron structure of the 3' portion of these two genes while the 5' structure of the genes diversified.

## Conclusions

In this study, we developed the CHAP package for detecting conversion events in gene clusters, and used this computational tool for analyzing five human gene clusters. We found that 20.0% of the paralogous sequence pairs in those clusters have undergone at least one conversion event. This is somewhat higher than the genome-wide frequency (13.5%) for all human paralog pairs [13], and confirms that conversion is a common phenomenon that must be accounted for when studying gene cluster evolution. Interestingly, some paralogs have experienced conversion events repeatedly in the same intervals; this can be detected if the later ones change narrower regions in the target.

To detect the occurrence of conversion events and their boundaries, we used a statistical test based on two criteria: the original triplet and quadruplet tests implemented in [12], and a new alternative test for events covering most or all of their respective paralogous sequences, which was implemented by extending the original detector. Both criteria achieve comparable statistical rigor by calculating a *P*-value for each event. We have compared our conversion detector pipeline (including the CAGE program) to other existing methods available for detecting conversion events, and the overall accuracy of our method (considering both the sensitivity and false discovery rate) was superior to the others when applied to gene cluster data [17]. The quality of the non-parametric statistical test used in our conversion detector was also evaluated in [18].

CHAP works best when sequences are available from many species, because even if a conversion cannot be detected using one particular primary or outgroup species (e.g. due to difficulty assigning orthologs), it may still be detected using others. In addition, using more species in the analysis enables more precise estimation of conversion times. However, as illustrated in the Drosophila example above, even one outgroup sequence is often sufficient to find conversions.

Accurate detection of conversion events can contribute substantially to other studies of gene cluster evolution by correcting duplication ages distorted by conversion and by improving the identification of orthology relationships in complex gene clusters. For example, the orthology assigned by CAGE is based on the positions where duplications occurred and the species in which they are seen (which we call "orthology by position"). The orthology mappings based on this definition are suitable for our purpose of conversion detection. However, other studies may require orthology mappings based on a different definition: if we trace the orthology based on the origin of the sequence contents rather than the positions of duplication, conversion events can change the mappings (for example, in Figure 2 the ortholog of A_2 _becomes B_1 _instead of B_2_). This alternative concept of "orthology by content" corresponds to the mosaic structures of phylogenetic trees [50], and is necessary for certain types of evolutionary analysis, such as determining the rate of synonymous substitutions in coding regions. In fact, it may not even be appropriate to analyze synonymous substitution rates in genes subject to frequent conversion events, because their nucleotide changes are not introduced according to a Poisson process, but rather as segments of changes. For both concepts of orthology, CHAP can lead to more accurate ortholog identification that is essential for constructing multiple sequence alignments and phylogenetic trees. Finally, our ongoing efforts in this area can help to more accurately model the full complexity of evolutionary processes, and to improve software for the analysis of gene cluster evolution.

## Methods

### Statistical triplet and quadruplet tests for conversion detection

The basic triplet test for conversion detection involves three homologous sequences: two paralogs from one species (A_1 _and A_2 _in Figure 2), and the (positional) ortholog of one of them in an outgroup species (say, B_1_). The alignments A_1_-A_2 _and A_1_-B_1 _are examined base-by-base using the hypergeometric random walk method of [18], which utilizes a cumulative score representing the difference in distances between these sequence pairs. This score is computed as follows: for each informative site in A1, the score increases if the paralog has the same nucleotide, but the ortholog has a different one, while the score decreases if the ortholog has the same nucleotide, but the paralog does not. (Note that the tree topology in Figure 2 is rooted, so sites can be informative with only three sequences involved. This enables us to detect conversion even when only one ortholog in the outgroup species is available, e.g. due to deletion of the other ortholog.) At the same time, we count the numbers of sites that increase and decrease the score, denoted as *m *and *n *respectively. When a maximum descent of *k *is observed for the hypergeometric random walk with *m *up and *n *down steps (*H*_*m,n*_), the *P*-value to determine the significance of the maximum descent is computed as the probability that the maximum descent of *H*_*m,n *_is greater than or equal to *k *by chance. When the *P*-value is below the cutoff threshold value, which is determined based on the multiple-comparison-corrected method proposed in [12], the interval of the maximum descent is taken as the converted region. See [12] for details, e.g. how to compute the *P*-value in a space- and time- efficient way in order to overcome the length limitation of ~400 informative sites encountered by [18].

Our detector from [12] also improves the method in another way, by using a quadruplet test where possible. If both paralogs (A_1 _and A_2_) have positional orthologs (B_1 _and B_2_, respectively), then the two corresponding triplet tests [A_1_, A_2_, B_1_] and [A_2_, A_1_, B_2_] are combined. At an informative site, if only one triplet shows an up step or down step, it is treated as usual. Otherwise, if both triplets show an up step for a column, we assign two up steps, and similarly if both show down steps (the combination of one up and one down step does not occur, because the two triplets have the same paralogous nucleotides). The maximum descent is determined based on the combined hypergeometric random walk. Then we use the same *P*-value formula as in the triplet test. This quadruplet test can improve the specificity of detection, because if one triplet falsely indicates a conversion event due to variation in evolutionary rate, it may be neutralized by the other triplet. Similarly, the quadruplet test can also enhance the sensitivity, because if a true conversion would have been missed due to weak support from one triplet, it may be corrected by strong support from the other one. In addition, the quadruplet test is often able to determine the direction of a conversion event by computing the probabilities of going down within the common maximum descent regions of the two triplets and determining the significance of the difference between the two probabilities based on a binomial distribution [12].

### Alternative criterion for conversions covering entire paralogous sequences

The paralogs for the triplet and quadruplet tests are obtained from intraspecies local alignments generated by LASTZ. Such alignments are highly sensitive to the parameter settings of the alignment program [51]. In addition, an intraspecies alignment tends to be less accurate than an interspecies one, due to the difficulty of computing alignment scores corresponding to the actual divergence time [52]. This is because each pair of paralogous regions can have its own unique divergence level according to their duplication time, whereas all of the orthologous pairs have been diverging over the same amount of evolutionary time. (Note that the LASTZ parameters and alignment scores used for this study are included as defaults in the CHAP package. They are recommended for conversion detection, but users can change these settings if desired.)

Alignment quality is especially problematic in the end regions of the intraspecies alignments, because (1) the ends of local alignments are generally defined by falling scores, and (2) the paralogs used by CHAP are entire local alignments, though their corresponding orthologs are usually excerpts from longer alignments. This increases the likelihood of calling false-positive conversions near the ends of the paralogs when using the original triplet and quadruplet tests. To avoid these spurious calls caused by erroneous end parts of intraspecies alignments, we skip these tests when the maximum descent covers almost the entire length of the paralogs (over 80%). We use 80% as the threshold because we observed that most conversion events are very short compared to the entire paralogous length, but the frequency of conversions called by the triplet/quadruplet tests increases drastically when the maximum descent covers more than 80% of the paralogous region. Note that the tests can handle the erroneous ends of intraspecies alignments better when the conversions are smaller, because informative sites in the non-converted regions reduce the effect of the erroneous ends of the paralogs. Small or medium-sized conversions near the end of a paralog pair may sometimes be reported as extending overly far into the poorly-aligning end region, but are much less likely to be completely spurious.

However, conversion events may sometimes genuinely cover more than 80% of their respective paralogs. In addition, the original triplet/quadruplet test causes false negatives for conversion events covering the entire paralogous alignments with no erroneous end parts, as well as false positives for erroneous alignments. When the maximum descent covers the entire paralogous length, the *P*-value computed by the original formula is equal to one due to lack of non-converted regions, undermining the significance of the event detection. Thus the original test is inadequate for detecting conversions covering entire or nearly entire paralogs. In order to avoid missing such events, we apply an alternative criterion utilizing the orthology mappings. When two paralogs show the descent pattern across more than 80% of their length, we distinguish conversion events from false positives according to whether or not the paralogs map to distinct ortholog regions in the outgroup species. If the two paralogs have distinct orthologs, we infer that they were formed by a duplication before the speciation. In this case a maximum descent covering nearly the entire paralog pair represents evidence for conversion, so we treat the descent region as a potential conversion event and calculate a *P*-value for its significance based on a binomial distribution. This test is inferior to the triplet/quadruplet test for typical conversions, but does not break down for the long ones. Other than this replacement of the *P*-value formula, all procedures for this alternative criterion are exactly the same as for the original triplet and quadruplet tests.

Suppose we know that the duplication occurred before the speciation event; then the probability of seeing an up step should be greater than the probability of seeing a down step, i.e., P(-1) < = 0.5. We further assume that the observations are independent. The probability of the number of down steps being > = *n *by chance (i.e. without conversion) can be calculated as

where *m *up and *n *down steps are observed and *p *= min(0.5, *n*/(*n + m*)). The parameter *p *is estimated from the observed number of up and down steps, and is at most 0.5.

### Redundant evidence for conversions

**Figure 13:**
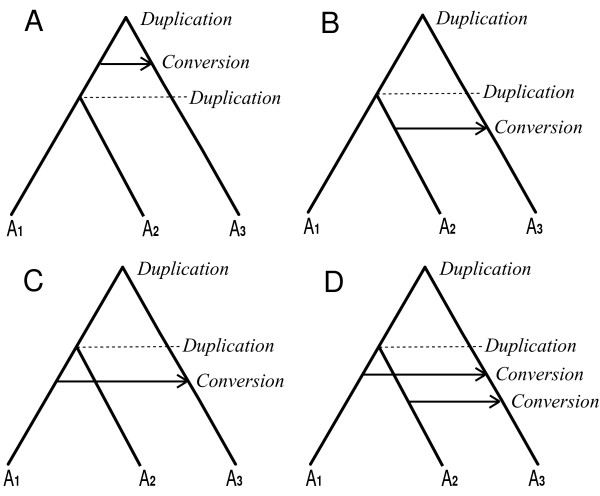
**Redundancy due to additional duplications**. The same conversion event may be detected using several paralog pairs in the same primary species. For instance in panel (A), A_3 _can be detected as the target of conversion from both A_1 _and A_2_. If the direction of the conversion was reversed, then both A_1 _and A_2 _could be detected as targets of conversion from A_3_. In (B) and (C), the new paralogs A_1 _and A_2 _may still be similar enough for A_3 _to be detected as the target of conversion from both, though only one was the actual source. But here if the direction of conversion was reversed there would be no confusion or redundancy, since the converted A_2 _would be easily distinguishable from the unconverted A_1 _(or vice-versa in (C)). In summary, the single conversions in (A)-(C) could be confused with case (D), which has two separate conversion events.

CHAP runs its tests for every combination of primary species, paralog pair in the primary species, and outgroup species. Because of this, it is common for a single conversion event to be detected more than once:

(a) If additional speciation events occurred between the duplication and the conversion, then the conversion may be detected using all of those outgroup species.

(b) If additional speciation events occurred after the conversion, then the conversion may be detected in all of the resulting primary species.

(c) If additional duplication events occurred after (or perhaps even slightly before) the conversion, then the conversion may be detected in all of the resulting paralogs (Figure 13).

CHAP's conversion detector watches for observations with similar boundaries that match these scenarios and assigns them the same event identifier in the output, as follows.

Type (a): For conversions detected using several outgroup species for a particular paralogous pair, the case of multiple observations for a single conversion event is distinguished from that of repeated conversion events by comparing the boundaries of the conversion regions. If their boundaries are equal or quite similar, we regard these multiple observations as representing the same conversion event. In the non-redundant version of the output, the observation with the smallest *P*-value is kept and the others are removed. Otherwise, they are considered to be separate, repeated conversion events.

Type (b): To remove redundancies in multiple primary species due to subsequent speciation, orthology information is needed. The CHAP pipeline obtains the orthologous relationships among all of the species using CAGE (Figure 3). In our procedure, if one conversion observation is detected between paralogs A_1 _and A_2 _in species A using outgroup species C, then all of the orthologous sequences of A_1 _and A_2 _in another primary species B, e.g. Orth(A_1_) = {B_11_, ..., B_1n_}, and Orth(A_2_) = {B_21_, ..., B_2m_}, are identified, and any conversion observations with similar endpoints between Orth(A_1_) and Orth(A_2_) which are detected using C as the outgroup species are assigned the same event id. This is repeated for all other species B, and in the non-redundant version of the output only one observation (the one with the lowest *P*-value) is kept for each event id.

Type (c): In Figure 13, two conversion observations, e.g. A_1_→A_3 _and A_2_→A_3_, are detected for four different cases. However, in the first three of these there is only one actual conversion event. In order to distinguish these four cases, we first check the boundaries of the conversion regions. If the boundaries are equal or quite similar, we regard them as one of the three cases in Figure 13A-C. Otherwise, the events are inferred to be separate, as in Figure 13D. In order to discriminate the first three cases in Figure 13A-C, the similarities between three pairs of sequences, S_1 _= Sim(A_1_, A_2_), S_2 _= Sim(A_1_, A_3_), and S_3 _= Sim(A_2_, A_3_), in the conversion regions are examined, where Sim(X, Y) is the similarity level (percentage identity) of regions X and Y based on their LASTZ alignment. The applicable case is determined as follows:

(1) If max(S_1_, S_2_, S_3_) = S_1_, then the conversion occurred before the duplication of A_1 _and A_2_, as shown in Figure 13A.

(2) If max(S_1_, S_2_, S_3_) = S_2_, then the conversion occurred between A_1 _and A_3_, as shown in Figure 13C.

(3) If max(S_1_, S_2_, S_3_) = S_3_, then the conversion occurred between A_2 _and A_3_, as shown in Figure 13B.

This is used to decide which observation is kept in the non-redundant version of the output; in case (1) the observation with the lowest *P*-value is used to represent the event, while in cases (2) and (3) the actual conversion is kept and the incorrect one removed.

The redundancies are removed in the order of Type (a), followed by (b), then (c). The order does not matter in principle, but may affect the results if some redundant observations are missing.

### Estimating the age of a conversion event

Redundancy information can be used as a guide to estimate the time of a conversion event, in terms of its placement on the species tree. Timing an event is composed of two parts. One is inferring an upper bound for the conversion time, i.e. its earliest possible branch edge in the tree, and the other is determining a lower bound.

An outgroup species used for detecting a conversion in a primary species helps to establish the upper bound, indicating that the conversion occurred after the separation of the primary and outgroup species. If an event in a primary species was detected using multiple outgroup species, the closest outgroup species to the primary one in the species tree is chosen to estimate the upper bound. When a conversion event was detected in multiple primary species, an upper bound for each primary species case is computed and then the most recent of these edges that is a common ancestor of all the primary species is assigned as the earliest possible conversion time.

In order to estimate the lower bound of the conversion time, a list of all primary species having the same conversion event is used. If we observed the same event in multiple primary species, that conversion predates the split of those primary species. So we infer the lower bound of the conversion time as the least common ancestral edge of those primary species in the species tree.

The conversion time is summarized as a path of possible edge numbers (sub-lineage) in the Branch column of Gmaj's table (Figure 7B). If a conflict is encountered when determining the conversion time due to an inconsistency in the data, then a question mark will appear in the output. However, this did not arise in our analysis of the five clusters.

### Simulation studies

CHAP's goal is to detect actual historical conversion events as accurately as possible. However it is also useful to have a more computational formulation of the problem, so that the general quality of the results can be assessed even though little is known about the true history of most clusters. Since we are developing the CHAP analysis package in conjunction with our gene cluster evolution simulator [17], the success of CHAP can be defined as the extent to which it can correctly recreate the known history of a simulated input sequence. However, we note that this framing of the problem shifts the burden of determining biological relevance onto the quality of the simulator, which currently emulates some, but not all, known evolutionary processes. Measuring the extent to which the simulated sequence reflects the characteristics of real-world gene clusters is a statistical problem that we leave for future work.

We conducted a study using our gene cluster evolution simulator from [17], in order to (1) more rigorously explore the effects of the main tuning parameter (the 80% paralog coverage threshold for criterion selection) on CHAP's sensitivity and false discovery rate, and to (2) measure CHAP's runtime performance on gene clusters of varying complexity. These results are available in the supplement (Additional file 1, Figure S1). We found that as expected, there is some trade-off between sensitivity and specificity of detection, but that the results are not greatly affected by the exact setting of this parameter. Our choice of 80% was initially selected by examining CHAP's output on real gene clusters for which some guidance is available in the literature regarding the actual converted regions. However, we found that 80% also looks reasonable, and arguably even optimal, on our simulated datasets where the true answers are known. We also observed that the runtime performance of our package is fairly fast (e.g. it finished the more complicated datasets within 15 minutes on average), despite using a probabilistic model.

## Authors' contributions

GS performed the experiments using the CHAP package. GS, CH, and CR implemented the pipeline. GS and CR wrote and finalized the manuscript. CH and YZ designed the statistical method for conversion detection and helped to write the Methods section. GS and HK analyzed the results. CR, FH, LZ, RH, EG, and WM helped with the analysis and writing. NISC Comparative Sequencing Program sequenced three new clusters in seven primates for this study. WM initiated, supervised, and coordinated the work. All authors read and approved the final manuscript.

## Supplementary Material

Additional file 1 **Supplement**. This includes Table S1 - GenBank accession numbers of the new sequences; Table S2 - Summary of detected conversions in the five human gene clusters; Table S3 - Fraction of paralogous pairs by their number of conversion events, out of all paralogous sequence pairs; Table S4 - Fraction of bases by their number of conversion events (involved as either source or target), out of all bases involved in duplications; Table S5 - Hot-spot segments of conversion events; and Figure S1 - Performance of our CHAP pipeline based on a simulation study. Click here for file
