# Supplementary material for: Conversion events in gene clusters
Source: BMC Evol Biol. 2011 Jul 28;11:226. doi: 10.1186/1471-2148-11-226 (PMC3161012; doi:10.1186/1471-2148-11-226)
Supplement: Additional file 1 — Supplement. This includes Table S1 - GenBank accession numbers of the new sequences; Table S2 - Summary of detected conversions in the five human gene clusters; Table S3 - Fraction of paralogous pairs by their number of conversion events, out of all paralogous sequence pairs; Table S4 - Fraction of bases by their number of conversion events (involved as either source or target), out of all bases involved in duplications; Table S5 - Hot-spot segments of conversion events; and Figure S1 - Performance of our CHAP pipeline based on a simulation study. [file 1471-2148-11-226-S1.PDF]

Supplementary information for

Conversion events in gene clusters

Table S1. GenBank accession numbers of the new sequences.

	CCL	IFN	CYP2abf
Gorilla	AC241834, AC239245, AC241429, AC233321	AC233322, AC233320, AC240917, AC237314, AC237197, AC241900, AC241430	DP001281
Colobus monkey	AC233589, AC233587, AC236260, AC236261	AC233327, AC233324, AC233326, AC237011, AC241433	DP001279
Vervet	AC240537, AC234054, AC234051	AC234658, AC234472, AC234007, AC235513, AC238663	DP001283
Dusky titi	AC234380, AC234273	AC233337, AC233338, AC234682, AC237242	DP001280
Spider monkey	AC241487, AC234683, AC234381	AC233249, AC233599, AC234821	DP001277
Black lemur	AC241901, AC236691, AC236693	AC237241, AC233592	DP001278
Lemur	AC239885, AC234681	AC236570	DP001282

Table S2. Summary of detected conversions in the five human gene clusters.

	β-globin	α-globin	CCL	IFN	CYP2abf	Total
Number of genes	5	5	13	17	6	46
Number of conversion events via criterion 1	9	6	16	91	36	158
Number of conversion events via criterion 2	2	5	8	62	21	98
Number of conversion events via both methods combined	11	11	24	153	57	256
Number of paralogous sequence pairs	29	18	123	644	161	975
Fraction of pairs showing conversion via criterion 1	24.14%	22.22%	8.13%	12.42%	18.63%	13.44%
Fraction of pairs showing conversion via criterion 2	6.90%	22.22%	6.50%	9.32%	12.42%	9.64%
Fraction of pairs showing conversion at least once	24.14%	38.89%	12.20%	19.25%	25.47%	19.90%

Total number of bases in duplications	12,461	11,900	290,624	204,100	206,609	725,694
Fraction of duplicated bases involved in conversion via criterion 1	45.18%	23.13%	8.91%	24.33%	24.05%	18.42%
Fraction of duplicated bases involved in conversion via criterion 2	58.51%	54.20%	10.19%	44.75%	33.57%	28.12%
Fraction of duplicated bases involved in conversion at least once	71.60%	75.03%	16.34%	50.86%	50.62%	37.72%
Number of coding exon bases	2,220	2,142	7,887	11,040	8,922	32,211
Fraction of coding bases involved in conversion via criterion 1	58.87%	1.82%	15.38%	64.63%	20.51%	35.78%
Fraction of coding bases involved in conversion via criterion 2	67.93%	51.45%	13.29%	72.11%	29.48%	44.24%
Fraction of coding bases involved in conversion at least once	73.78%	51.45%	22.68%	72.82%	42.21%	50.71%

Criterion 1 signifies events detected by the original triplet or quadruplet tests in Hsu et al. (2010), while criterion 2 is our new complementary method for detecting conversion events covering most or all of an entire paralog. Bases involved in conversion include both donor and recipient regions.

Table S3. Fraction of paralogous pairs by their number of conversion events, out of all paralogous sequence pairs.

	β-globin	α-globin	CCL	IFN	CYP2abf
0	75.86%	61.11%	87.80%	80.75%	74.53%
1	13.79%	27.78%	7.32%	15.53%	16.77%
2	6.90%	0.00%	3.25%	3.11%	7.45%
3	3.45%	11.11%	0.81%	0.47%	1.24%
>=4 events	0.00%	0.00%	0.81%	0.16%	0.00%

Table S4. Fraction of bases by their number of conversion events (involved as either source or target), out of all bases involved in duplications.

	β-globin	α-globin	CCL	IFN	CYP2abf
0	28.40%	24.97%	83.66%	49.14%	49.38%
1	39.23%	51.40%	12.00%	23.80%	39.12%
2	31.32%	9.54%	3.20%	9.87%	9.70%
3	1.00%	13.98%	0.81%	6.54%	1.35%
>=4 events	0.05%	0.10%	0.34%	10.65%	0.45%

Table S5. Hot-spot regions for conversion events. We partitioned each gene cluster into segments using breakpoints from all conversion events, and identified the one showing the most conversion events in each cluster as a "hot-spot" segment. In CCL there was a tie, with four segments having four events each.

Cluster	Chromosome	Start	End	Length	Annotation	Number of events
β-globin	chr11	5247854	5247859	6	second exon in HBB	4
α-globin	chr16	204195	204206	12	intron in HBZ	4
CCL	chr17	34325584	34325679	96	intron in CCL15	4
	chr17	34341056	34341151	96	intron in CCL23	4
	chr17	34398281	34398353	73	intron and exon in CCL18	4
	chr17	34410352	34411081	730	no gene	4
IFN	chr9	21239532	21239577	46	exon in IFNA14	13
CYP2abf	chr19	41530849	41531369	521	no gene	5

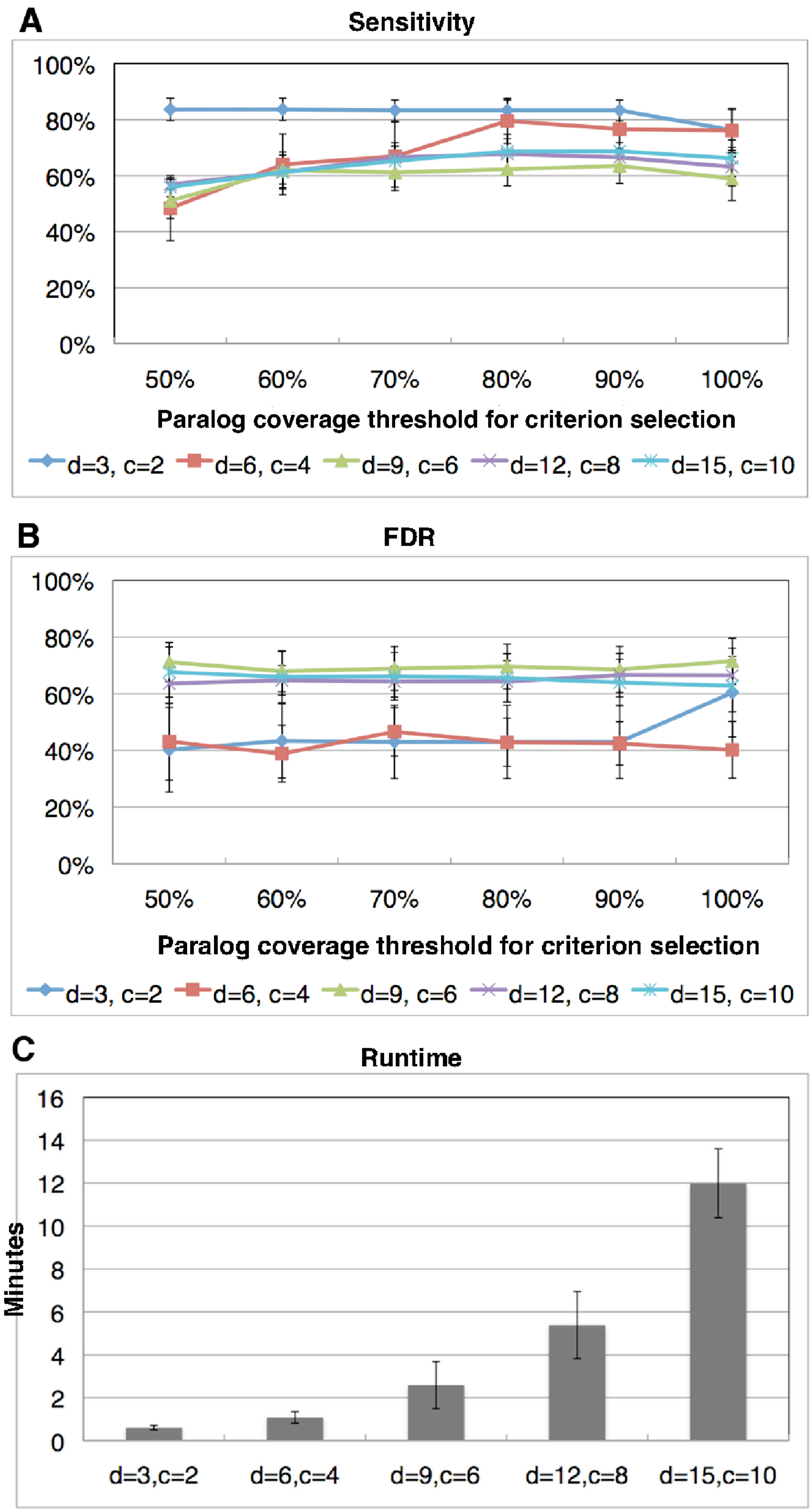
A
Sensitivity
100%
80%
60%
40%
20%
0%
50%
60%
70%
80%
90%
100%
Paralog coverage threshold for criterion selection
d=3, c=2
d=6, c=4
d=9, c=6
d=12, c=8
d=15, c=10
B
FDR
100%
80%
60%
40%
20%
0%
50%
60%
70%
80%
90%
100%
Paralog coverage threshold for criterion selection
d=3, c=2
d=6, c=4
d=9, c=6
d=12, c=8
d=15, c=10
C
Runtime
Minutes
16
14
12
10
8
6
4
2
0
d=3,c=2
d=6,c=4
d=9,c=6
d=12,c=8
d=15,c=10

Figure S1. Performance of our CHAP pipeline based on a simulation study.
Simulated sequences mimicking gene clusters in humans, old world monkeys (OWM), and new world monkeys (NWM) were generated using our method from Song et al. (2011), which starts with a 200 kb duplication-free "ancestral" sequence and emulates duplication, conversion, speciation, small substitutions, and purifying selection. Each simulation run included *d* duplications in the ancestral lineage of the three clades, another *d* duplications plus *c* conversions in each lineage between the splits of NWM and OWM from the human lineage, and still another *d* duplications and *c* conversions in each lineage after the split of humans and OWM, for d={3, 6, 9, 12, 15} and c={2, 4, 6, 8, 10} respectively. We generated five replications for each level of evolutionary complexity. Then we ran the CHAP pipeline to detect conversion events in each simulated dataset for values of the criterion selection threshold ranging from 50-100%, and compared its output to the true events known from the simulation process. The results are shown in three plots for (A) sensitivity: the fraction of converted bases detected correctly, (B) FDR (false discovery rate): the fraction of called bases detected incorrectly, and (C) CHAP's run time on a Linux 2.6.18 machine with a 8-core Intel Xeon CPU (2826 MHz) and 32 GB of RAM memory. Error bars indicate the standard deviation of the replications at each point.

Reference

1. Hsu C, Zhang Y, Hardison R, NISC Comparative Sequencing Program, Green E, Miller W: **An effective method for detecting gene conversion events in whole genomes.** *J Comput Biol* 2010, **17**: 1281–1297.
2. Song G, Hsu C, Riemer C, Miller W: **Evaluation of methods for detecting conversion events in gene clusters.** *BMC Bioinformatics* 2011, **12**(Suppl 1):S45.
